# *Kerstersia gyiorum* Isolated from a Bronchoalveolar Lavage in a Patient with a Chronic Tracheostomy

**DOI:** 10.1155/2014/479581

**Published:** 2014-11-23

**Authors:** Meredith Deutscher, Jennifer Severing, Joan-Miquel Balada-Llasat

**Affiliations:** ^1^Division of Infectious Diseases, Kaiser Permanente Medical Center, Oakland, CA 94611, USA; ^2^Pharmacy Department, The Ohio State University Wexner Medical Center, Columbus, OH 43205, USA; ^3^Department of Pathology, The Ohio State University Wexner Medical Center, University Hospital East, 1492 East Broad Street, Columbus, OH 43205, USA

## Abstract

The use of the matrix-assisted laser desorption ionization-time of flight (MALDI-TOF) mass spectrometry (MS) generates rapid microbial identification. We are presenting a case of a 63-year-old woman with a medical history of chronic tracheostomy admitted for hypotension and fevers to illustrate the clinical implication of MALDI-TOF MS on bacterial identification. *Kerstersia gyiorum* was identified from the bronchoalveolar lavage isolate. *Kerstersia gyiorum* has been isolated from human sputum samples, and may be a previously unrecognized colonizer of the upper respiratory tract. Thus, patients with long-term tracheotomies or who are chronically aspirating may be at risk of lower respiratory infection with this organism. Increased use of MALDI-TOF MS in the clinical setting may increase reporting of this atypical isolate.

## 1. Introduction

MALDI-TOF MS provides accurate identification that compares well with that of genomic sequencing. Moreover, the MALDI-TOF MS is quicker, providing identification within 1-2 hours compared to traditional methods or sequencing that may take days for definitive identification. We report isolation and identification of* Kerstersia gyiorum* by MALDI-TOF MS from a bronchoalveolar lavage isolate in a patient with a chronic tracheostomy. This case confirms the use of MALDI-TOF MS as a reliable technique for the accurate identification of* Kerstersia gyiorum* and illustrates the impact of MALDI-TOF MS on patient management.

## 2. Case Report

A 63-year-old woman with a medical history of ventilator-dependent chronic respiratory failure, end-stage renal disease requiring dialysis, and residual left-sided weakness secondary to a remote stroke was transferred from an outpatient dialysis center to the Emergency Department at The Ohio State University Wexner Medical Center for evaluation of a dialysis catheter site infection. The patient developed hypotension and fevers during her dialysis session, and upon examination at the dialysis unit the dialysis catheter insertion site had purulent discharge. She already had received two weeks of vancomycin for a reported methicillin-resistant* Staphylococcus aureus* bacteremia and was given an additional dose of vancomycin before transferring to the emergency department. The dialysis catheter was removed on Day 1 of hospitalization, and cultures from the tip grew >15 colony forming units/mL* S. aureus* and <15 colony forming units/mL* Proteus*-like organisms. Blood cultures obtained in the emergency department were negative after five days, and the patient completed a 14-day course of daptomycin.

At the time of admission, the patient was also diagnosed with tracheobronchitis and was prescribed empiric piperacillin-tazobactam. Tracheal aspirate obtained on Day 1 of hospitalization grew* Pseudomonas aeruginosa, Providencia stuartii*, and* Proteus mirabilis*. When* P. mirabilis* was confirmed to be an extended-spectrum beta lactamase producer, piperacillin-tazobactam was discontinued and doripenem was prescribed. On Day 10 the patient suddenly developed increased FiO_2_ requirements, increased purulent tracheal secretions, and rhonchi on respiratory examination. Mini bronchoalveolar lavage was performed. The direct Gram stain of the specimen showed moderate polymorphonuclear cells and intracellular Gram-negative bacilli. After 24-hour incubation at 37°C and 5% CO_2_, three different colony types grew on blood and chocolate agars (Thermo Fisher Scientific Remel Products, Lenexa, KS).* P. aeruginosa* and* Stenotrophomonas maltophilia* were identified by MALDI-TOF MS using the BioTyper system (software version 3.1; Bruker-Daltonics, Billerica, MA). Ciprofloxacin and ceftazidime were added to cover* S. maltophilia* (the patient was allergic to sulfonamides).* Pseudomonas aeruginosa* was only susceptible to tobramycin, amikacin, and colistin, and the patient was prescribed intravenous and inhaled colistin to treat this pathogen.

**Figure 1:**
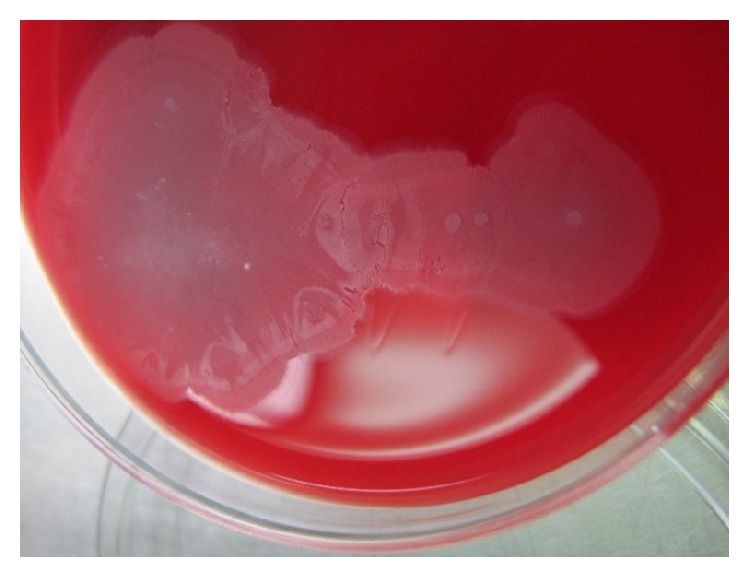
*K. gyiorum* appear as gray colonies with spreading edges on blood agar.

**Figure 2:**
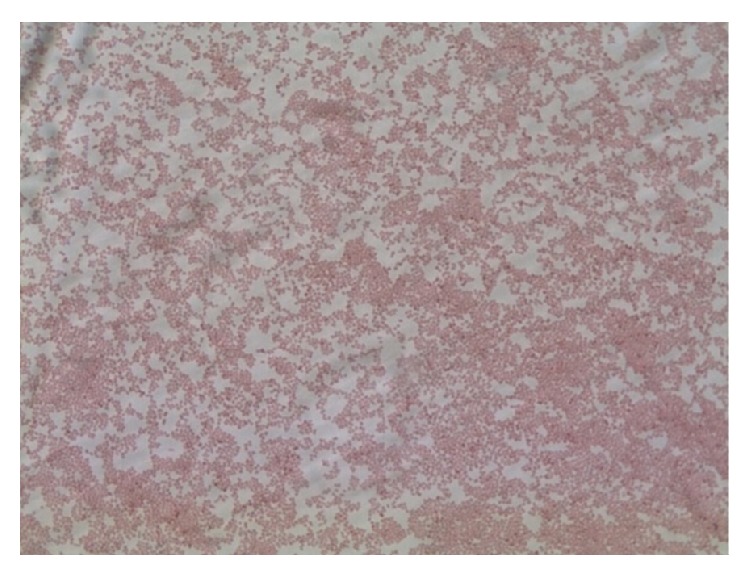
Gram-negative coccobacilli (1000x).

The third colony type showed spreading edge morphology (Figure 1). A Gram stain of the colony showed Gram-negative coccobacilli (Figure 2). The isolate was oxidase negative and catalase positive. Per institution protocol for respiratory isolates, the isolate was analyzed by MALDI-TOF MS and was identified as* Kerstersia gyiorum*. A BioTyper score of 2.3 (excellent identification) was obtained with a formic acid overlay. The identification of* K. gyiorum* by MALDI-TOF MS was confirmed by 16S rRNA gene sequencing using previous described methodology [1, 2].

Susceptibility to seven antimicrobial agents was determined by the Etest method (bioMérieux, Marcy l'Étoile, France) on Mueller-Hinton agar. The MIC breakpoints were those established by the Clinical and Laboratory Standards Institute for other non-Enterobacteriaceae [3]. The isolate was susceptible to gentamicin (≤4 *μ*g/mL), amikacin (≤16 *μ*g/mL), tobramycin (≤4 *μ*g/mL), cefepime (≤4 *μ*g/mL), piperacillin/tazobactam (≤16 *μ*g/mL), and trimethoprim/sulfamethoxazole (≤2/38 *μ*g/mL). The MIC of ciprofloxacin was 2 *μ*g/mL (intermediate).

The patient's fevers and copious sputum production resolved on the adjusted antibiotic regimen and she was transferred to a subacute care hospital. She was transferred back the next day secondary to persistent hypotension requiring vasopressors. The family requested that a Do Not Resuscitate Comfort Care protocol be initiated and, with the exception of mechanical ventilation, that life support be withdrawn. The patient died on Day 5 of her second hospitalization.

## 3. Discussion

This is the first paper describing* K. gyiorum* isolated from the lower respiratory tract.* Kerstersia* spp. are a member of the family Alcaligenaceae and are closely related to* Alcaligenes, Bordetella*, and* Achromobacter* spp., although it is oxidase negative, in contrast to the other genera [4].* K. gyiorum* was initially described by Coenye et al. in 2003 using isolates from human feces, leg wounds, and sputum [4]. There are only a few case reports documenting* Kerstersia* spp. infection. Almuzara et al. described a case of* K. gyiorum* isolated from a patient with cholesteatomatous chronic otitis media [5]. A second species of* Kerstersia, Kerstersia similis*, also was identified from this clinical specimen. Vandamme et al. reported that* K. similis* was isolated from a neck abscess and leg wound [6]. Pence et al. described two cases of* K. gyiorum*, one isolated from a patient with chronic ear infections and the other from a patient with a chronic leg wound [7]. Like these cases,* K. gyiorum* in our patient was isolated in the setting of a longstanding inflammatory condition, chronic respiratory failure.

As other bacterial species were also isolated in the specimen, it is difficult to know how much of the disease process can specifically be attributed to* K. gyiorum*, but we suspect that the patient's worsening respiratory status can be attributed to infection with* P. aeruginosa* and* S. maltophilia*.* Kerstersia* spp. have been isolated from human sputum samples and may be a previously unidentified colonizer of the upper respiratory tract. Thus, patients with long-term tracheotomies or who are chronically aspirating may be at risk of lower respiratory infection with this organism.

There are only two prior descriptions of this species exhibiting a spreading edge morphology on blood and chocolate agar [7], but based on these three cases, these phenotypes may be characteristic of* K. gyiorum*. The spreading morphology distinguishes* K. gyiorum* from* Acinetobacter* spp., which are also oxidase negative, nonfermenting Gram-negative bacilli. With relation to the antibiotic sensitivity, our isolate was susceptible to aminoglycosides, to ciprofloxacin, and to broad-spectrum cephalosporins, results which are in agreement with those published by Coenye et al. [4].

As noted by Pence et al., because of the unusual nature of these isolates, it is unlikely that we would have been able to assign an identification solely using biochemical methods.* K. gyiorum* is not included in the databases of most commercial systems, and thus, MALDI-TOF MS or sequencing techniques need to be used [7]. In an effort to expedite timely, effective antimicrobial therapy for our patients with pneumonia, our institution implemented MALDIT-TOF MS testing for all respiratory isolates in June 2012. As identification methods such as MALDI-TOF MS and 16S rRNA gene sequencing become more widely adopted in clinical laboratories, an increasing number of atypical organisms are likely to be identified.
